# The influence of closure technique in total laryngectomy on the development of a pseudo-diverticulum and dysphagia

**DOI:** 10.1007/s00405-016-4424-4

**Published:** 2016-12-21

**Authors:** Martine F. van der Kamp, Rico N. P. M. Rinkel, Simone E. J. Eerenstein

**Affiliations:** Department of Otolaryngology-Head and Neck Surgery, VU Medical Centre, Amsterdam, The Netherlands

**Keywords:** Oncology, Head and neck surgery, Laryngectomy, Suture methods, Pseudo-diverticulum, Dysphagia

## Abstract

**Objective:**

In total laryngectomy, the neopharynx can be closed in several ways. It is suggested that a pseudo-diverticulum is seen more frequently in patients closed with vertical closure than with “T”-shaped closure, causing postoperative dysphagia. We report the results of patients treated with vertical closure and “T”-shaped closure with regard to the formation of a pseudo-diverticulum and postoperative dysphagia.

**Methods:**

In our retrospective cohort study, we identified 117 consecutive laryngectomized patients treated in the VU University Medical Center of Amsterdam between March 2009 and December 2013. Evaluations with statistical analysis of postoperative outcome measures (the formation of a pseudo-diverticulum and dysphagia), qualitative and quantitative variables were conducted.

**Results:**

Patient demographics were similar between the vertical-shaped closure and the “T”-shaped closure groups. In 84.6% of patients with vertical closure, a pseudo-diverticulum was seen compared to 18.5% with “T”-shaped closure (p < 0.001). Dysphagia was increasingly seen in patients with a pseudo-diverticulum (60.5%) compared to patients without a pseudo-diverticulum (39.5%) (p = 0.090).

**Conclusion:**

Formation of a pseudo-diverticulum is more frequently seen in laryngectomy patients closed with vertical closure than in patients closed with “T”-shaped closure of the neopharynx. It is favorable to implement “T”-shaped closure in laryngectomy.

## Introduction

**Fig. 1:**
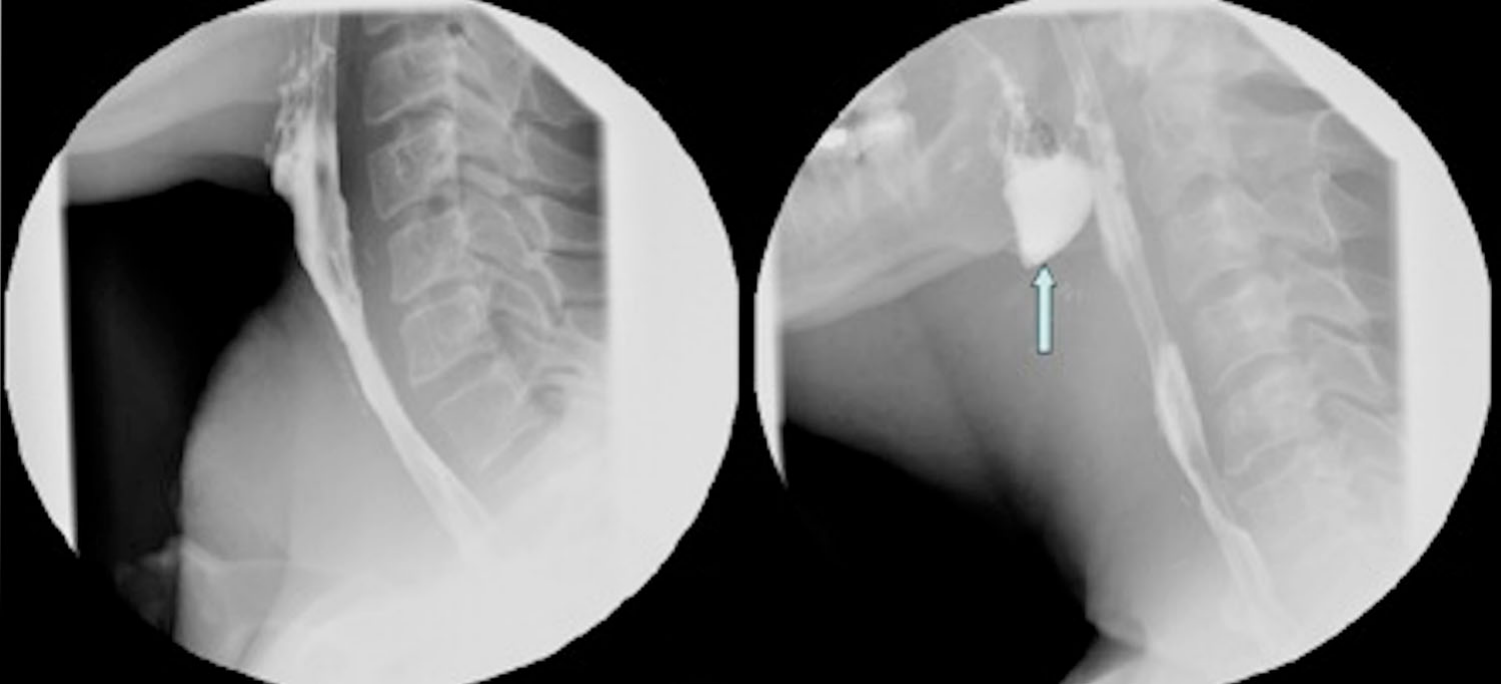
Barium swallow radiograph of a patient without a pseudo-diverticulum (**a**) and of a patient with a pseudo-diverticulum (*arrow*) (**b**) after laryngectomy

One of the treatment options in advanced stage laryngeal and hypopharyngeal cancer is laryngectomy. In total laryngectomy, with or without pharyngectomy, the larynx and potential part of the anterior pharyngeal mucosa are removed [1]. A tracheostomy is formed and the resulting surgical defect on the anterior site of the pharynx is closed, creating the so-called neopharynx [2]. The most common surgical techniques for primary closure of the neopharynx are “T”-shaped closure or vertical closure of the pharyngeal tissue. The choice of surgical technique is based on surgeons’ and institute’s preference. Dysphagia is one of the most common and well-known postoperative symptoms in patients after laryngectomy [3, 4]. The reported incidence of postoperative dysphagia varies from 17 to 72% [1, 5, 6]. This symptom has several causes, such as tumour recurrence, pharyngeal dysmotility, stricture formation, pharyngocutaneous fistulas, and postoperative radiotherapy but also the formation of a pseudo-diverticulum [7–9]. The pseudo-diverticulum is an anteriorly located mucosalized pouch of the neopharyngeal lumen, situated at the base of the tongue (Fig. 1) [7, 10, 12]. The kind of dysphagia that it can cause can be obstructive, the feeling of having to “swallow over a hump” or with a typical regurgitative character similar to the dysphagia found in patients with a Zenker’s diverticulum [10]. History of these symptoms combined with physical examination and/or barium swallow radiograph leads to the diagnosis of the pseudo-diverticulum.

The pseudo-diverticulum might be an overlooked cause for postoperative dysphagia [11, 13]. According to relevant literature the incidence ranges from 35 to 86% [7–9, 13, 14]. Although the possible association between closure technique in laryngectomy, pseudo-diverticulum and postoperative dysphagia has been described before, little scientific research has been published on this topic. Only one study from Davis et al. described an association between vertical closure technique and the formation of a pseudo-diverticulum in 1982 [13]. The aim of our study was to determine whether technique of surgical pharyngeal closure (vertical vs. “T”-shaped closure) is associated with the formation of a pseudo-diverticulum and whether this pseudo-diverticulum is associated with postoperative symptoms of dysphagia. We also determined if type of closure was associated with postoperative fistula formation.

## Materials and methods

We conducted a retrospective cohort study in consecutive laryngectomy patients admitted to the VU University Medical Center Amsterdam between March 2009 and December 2013. Patients were excluded from analysis when they had any type of donor reconstruction of the neopharynx, for example, pectoralis major or free flap reconstruction. Also patients without postoperative barium swallow radiograph or with a barium swallow radiograph of poor quality were excluded. Postoperative follow-up ranged from seven months to five and a half years.

### Outcome parameters

The primary outcome parameter was the formation of a pseudo-diverticulum. The secondary outcome parameter was postoperative dysphagia. Additionally we examined fistula formation for secondary analysis.

### Data

Surgical records were examined for details about the technique of mucosal closure—vertical or “T”-shaped.

**Fig. 2:**
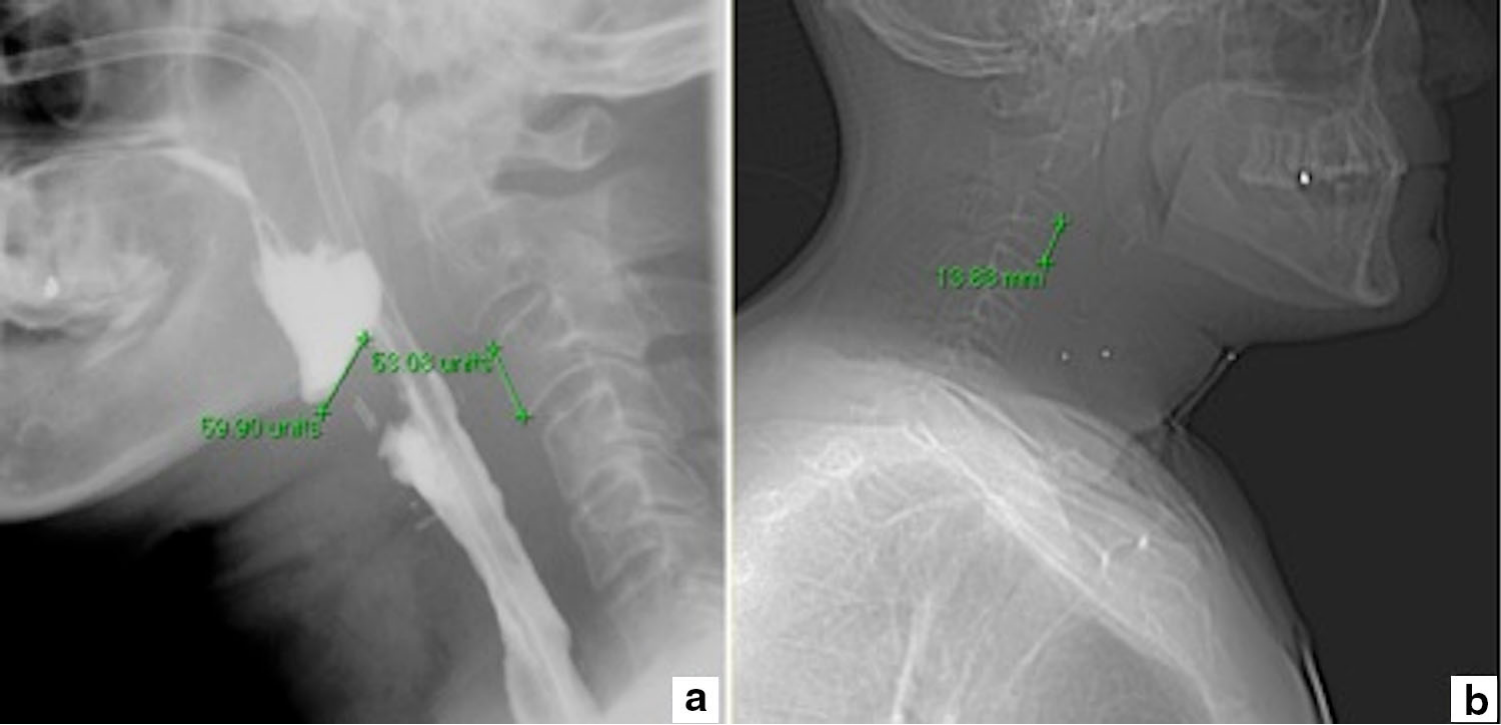
Measurement of the pseudo-diverticulum and cervical vertebra C3 in barium swallow radiograph after laryngopharyngectomy (**a**), and measurement of cervical vertebra C3 in CT-scan (**b**)

Information regarding postoperative dysphagia was obtained from postoperative completed validated questionnaires, the SWAL-QOL, and from the patients’ medical files [15]. Dysphagia was scored as “present” when a patient’s total score on the SWAL-QOL was above the cut-off point (≥14 points) [15]. The minimum score of the SWAL-QOL is 0 points and the maximum score is 120 points. When the SWAL-QOL was not completed, dysphagia was defined as any evident swallowing dysfunction recorded in the patient’s medical file. Barium swallow radiographs—made between 10 and 14 days postoperatively—from all patients were scored. Two of the authors scored the presence of a pseudo-diverticulum and measured its depth, both blinded for type of closure and dysphagia. A pseudo-diverticulum was scored when on sagittal projection an anteriorly located tissue-bar with an out-pouching of the neopharynx was observed and a certain amount of contrast remained in this out-pouch after swallowing (Fig. 1). Subsequently, the deepest size of the pseudo-diverticulum was measured. Measurements in barium swallow radiographs could only be performed in units. Units were converted to millimetres by measuring the height of the third (or when not applicable, the second) cervical vertebra in units on the barium swallow radiograph and of the same vertebra in millimetres on CT-scan or MRI-scan (Fig. 2). Additionally, postoperative fistulas were identified, defined as any evident visible leakage on imaging. Medical records were used to retrieve demographical and clinical variables that may confound the association between the formation of a pseudo-diverticulum and technique of surgical closure. Variables that might cause wound-healing problems and therefore could induce the risk for developing a pseudo-diverticulum were used, such as risk factors for head and neck squamous cell carcinomas (tobacco use and alcohol intake, tobacco use scored in pack years, and alcohol intake in units per day at time of laryngectomy) [16], adjuvant therapy (preoperative chemo- and radiotherapy), comorbidities (ASA-score) [17], postoperative complications (i.e., wound infections, fistulas, chyle leakage, stricture formation, and haemorrhage) and pre- and postoperative albumin and haemoglobin value. Other factors that could possibly induce the risk for developing a pseudo-diverticulum or dysphagia were several tumour details such as location, stage (TNM staging), primary or recurrent tumour, histopathological stage, and history of previous head- and neck carcinoma.

### Statistical analysis

Statistical analyses were performed using SPSS (version 20, IBM Corp., Armonk, NY). Baseline characteristics and clinical variables were compared between the two closure methods and presented as mean and standard deviation (SD), median and range or frequencies. Independent sample *t* test (normal data), Mann–Whitney test (non-normal data) and the Chi-square test (categorical data) were used. Variables with *p* value <0.05 were considered potential confounders and further analysed via multivariable logistic regression analysis. A *p* value <0.05 was considered statistically significant, a *p* value <0.1 was considered to indicate a trend.

## Results

### Patients’ characteristics

A total of 112 patients were included in our consecutive cohort of laryngectomies. For several reasons, we were obliged to exclude some patients of this certain cohort; 37 patients had a donor flap reconstruction of the neopharynx instead of primary pharyngeal closure, in five patients postoperative barium swallow radiograph was not performed or was of poor quality and in four patients the operation report was missing. This resulted in 66 patients eligible for final analysis.

Cohort information is given in Table 1. In 39 of the 66 patients, vertical closure was performed and in 27 “T”-shaped closure was performed. Mean age in the vertical closure group was 64 years (SD 10.1) and in the “T”-shaped closure group 61 years (SD 10.6). 34 (87.2%) of the patients in the vertical closure group were men and 22 (81.5%) in the “T”-shaped closure group. Timing of adjuvant therapy varied, 19 (48.7%) patients in the vertical closure group underwent preoperative radiotherapy compared to 9 (33.3%) in the “T”-shaped closure group. Preoperative chemotherapy was given to 5 (12.8%) patients in the vertical closure group compared to 1 (3.7%) patient in the “T”-shaped closure group. Median preoperative albumin value was 34 g/L equal in both groups. In the vertical closure group, the median of preoperative haemoglobin was 8.3 mmol/L (range 5.2–9.7) compared to 8.6 mmol/L (range 6.6–10.3) in the “T”-shaped closure group. The vertical closure group contained 3 (7.7%) hypopharyngeal and 35 (89.7%) laryngeal tumours compared to 1 (3.7%) hypopharyngeal tumour and 26 (96.3%) laryngeal tumours in the “T”-shaped closure group. The vertical closure group showed higher numbers of patients with a primary tumour, with 19 (48.7%) patients having a primary tumour and 20 (51.3%) patients having a recurrent tumour. In the “T”-shaped closure 20 (74.1%) patients had a primary tumour and 7 (25.9%) had a recurrent tumour. In both groups, high TNM stages were seen, 24 (61.5%) patients had T3–T4 staged tumours with 18 (46.2%) patients with positive lymph nodes in the vertical closure group compared to 18 (66.7) patients with T3–T4 staged tumours and 10 (37.0%) patients with positive lymph nodes in the “T”-shaped closure group. Comorbidities were scored as I. no comorbidities, II. mild disease and III. severe disease. In the vertical closure group, respectively, 3 (7.7%), 23 (59.0%) and 13 (33.3%) and in the “T”-shaped closure group, respectively, 1 (3.7%), 11 (40.7%), and 15 (55.6%) patients were scored. In 22 (56.4%) patients in the vertical closure group varying degrees of complications were seen (i.e., fistulae, wound infections, and haemorrhage) compared to 14 (51.9%) patients in the “T”-shaped group.

**Table 1:** Patient characteristics for the two types of closure

	Vertical closure *n* = 39	“T”-shaped closure *n* = 27	*p* value
Age, mean (SD)	64.0 (10.1)	60.9 (10.6)	0.26
BMI, mean (SD)	34.3 (4.4)	24.9 (4.8)	0.37
Tobacco use*, median (range)	36.5 (0–110)	36.8 (0–120)	0.70
Alcohol use (i.e./day), median (range)	2.0 (0–40)	1.0 (0–20)	0.16
Albumin preop (g/L), median (range)	34.0 (27–63)	34.0 (22–42)	0.39
Hb preop (millimol/L), median (range)	8.3 (5.2–9.7)	8.6 (6.6–10.3)	0.75
Sex			0.53
Male	34 (87.2%)	22 (81.5%)	
Female	5 (12.8%)	5 (18.5%)	
Tumour site			0.64
Hypopharynx	3 (7.7%)	1 (3.7%)	
Larynx	35 (89.7%)	26 (96.3%)	
Other	1 (2.6%)	0	
Histopathological			0.82
SCC	34 (87.2%)	23 (85.2%)	
Other	5 (12.8%)	4 (14.8%)	
Type of tumour			0.039
Primary	19 (48.7%)	20 (74.1%)	
Recurrence	20 (51.3%)	7 (25.9%)	
T-stage			1.00
T1 + T2	4 (10.3%)	2 (7.4%)	
T3 + T4	24 (61.5%)	18 (66.7%)	
Unknown	11 (28.2%)	7 (25.9%)	
N-stage			0.31
N-positive	18 (46.2%)	10 (37.0%)	
N-negative	11 (28.2%)	11 (40.7%)	
Unknown	10 (26.3%)	6 (22.2%)	
Preop RT			0.21
Yes	19 (48.7%)	9 (33.3%)	
No	20 (51.3%)	18 (66.7%)	
Preop CT			0.39
Yes	5 (12.8%)	1 (3.7%)	
No	34 (87.2%)	26 (96.3%)	
Comorbidities^a^			0.080
I. No	3 (7.7%)	1 (3.7%)	
II. Mild	23 (59.0%)	11 (40.7%)	
III. Severe	13 (33.3%)	15 (55.6%)	
Complications			0.72
Yes	22 (56.4%)	14 (51.9%)	
No	17 (43.6%)	13 (48.1%)	

*BMI* body mass index, *preop* preoperative, *Hb* haemoglobin, *SCC* squamous cell carcinoma, *RT* radiotherapy, *CT* chemotherapy

*p* < 0.05 was considered significant

* Packyears

^a^ASA-score

### Pseudo-diverticulum

A pseudo-diverticulum was seen significantly more often in patients with a vertically closed neopharynx (*n* = 33, 84.6%) compared to patients with “T”-shaped closure of the neopharynx (*n* = 5, 18.5%; *p* < 0.001) (Table 2). In the vertical closure group, the median size of the pseudo-diverticulum was 14.8 mm (range 4–26 mm) compared to 12.9 mm (range 5–20 mm) in the “T”-shaped closure group (Table 2). Univariate analyses with respect to closure technique identified only one potential confounder, ‘Type of tumour’, in the association between formation of the pseudo-diverticulum and closure technique. However, since ‘Type of tumour’ was not associated with the formation of a pseudo-diverticulum, it is not considered a confounder. SWAL-QOL total scores did not differ significantly between patients with or without a pseudo-diverticulum (*p* = 0.57).

**Table 2:** Results pseudo-diverticulum, size, dysphagia, and fistula

	Vertical closure *n* = 39	“T”-shaped closure *n* = 27	*p* value
Pseudo-diverticulum			<0.001
Yes	*n* = 33 (84.6%)	*n* = 5 (18.5%)	
No	*n* = 6 (15.4%)	*n* = 22 (81.5%)	
Size PD*, median (range)	14.8 (4–26)	12.9 (5–20)	0.33
Dysphagia			0.65
Yes	*n* = 21 (53.8%)	*n* = 13 (48.1%)	
No	*n* = 18 (46.2%)	*n* = 14 (51.9%)	
SWAL-QOL total score	*n* = 11	*n* = 12	0.51
Mean (SD)	32	46	
Range	7–50	21–90	
Fistula			
Yes	*n* = 9 (23.1%)	*n* = 4 (14.8%)	0.40
No	*n* = 30 (76.9%)	*n* = 23 (85.2%)	

*PD* pseudo-diverticulum

*p* < 0.05 was considered significant

* Millimetres

### Dysphagia

Twenty-three (60.5%) patients with a pseudo-diverticulum had symptoms of dysphagia compared to 15 (39.5%) patients without a pseudo-diverticulum. This difference was not statistically significant but indicative of a statistical trend (*p* = 0.088). No difference was found between the type of closure and postoperative dysphagia either and there was no significant difference between the SWAL-QOL total score in both groups (*p* = 0.51) (Table 2).

### Fistula

No association was found between type of closure and postoperative fistulas. Postoperative fistulas were seen in 9 (23.1%) of the vertically closed patients compared to 4 (14.8%) of the “T”-shape closed patients (*p* = 0.40) (Table 2).

## Discussion

Dysphagia is one of the most common and well-known symptoms seen in laryngectomized patients. One of the etiologic sources for this postoperative dysphagia is the pseudo-diverticulum. Commonly used text books such as Stell & Maran’s textbook of Head and Neck Surgery and oncology suggest that the formation of a pseudo-diverticulum is more often related to patients who have had vertical closure of the neopharynx during laryngectomy instead of “T”-shaped closure [12]. However, little scientific research has been done about the influence of type of closure of the neopharynx on the development of this pseudo-diverticulum and thereby postoperative dysphagia. In the current standards, the preference of the surgeon decides what type of closure is performed during laryngectomy surgery. To determine whether there is a type of surgical closure of the neopharynx leading to the formation of a pseudo-diverticulum and postoperative dysphagia, this study aimed to compare two closure techniques: vertical closure versus “T”-shaped closure. The present study showed that the formation of a pseudo-diverticulum is more often seen in patients with vertical closure of the neopharynx than with “T”-shaped closure in laryngectomy. A trend (*p* = 0.088) for lower prevalence of postoperative dysphagia was reported in patients without a pseudo-diverticulum compared to patients with a pseudo-diverticulum. In our opinion, the theory behind this problem is that when applying vertical closure, a surplus of tissue is formed that is creating the pseudo-diverticulum. When applying “T”-shaped closure, there will be less surplus and this surplus will be fixed to the base of the tongue, which could prevent for the formation of a pseudo-diverticulum. Our study showed that type of closure was not associated with the formation of fistulas or other postoperative complications. In our vision, applying “T”-shaped closure instead of vertical closure in laryngectomy may result in reducing the risk for developing a pseudo-diverticulum and probably postoperative dysphagia. In 1982, Davis et al. described an association between the type of closure and the presence and absence of a “pseudo-epiglottis” (a fold of mucous membrane and sometimes scar tissue coming from the anterior pharyngeal wall into the base of the tongue, superior to the pseudo-diverticulum), in a small study with 20 patients [13]. All vertically closed patients (*n* = 11) had a pseudo-epiglottis compared to 67% (*n* = 6) patients closed with “T”-shaped closure (*p* < 0.05). In this study, one explanation for these results was that tension on the wound edges is caused by contraction of the tongue muscles in one direction and the pharyngeal constrictors in the opposite direction. Another explanation was that after vertical closure the tension on the tongue is released, which causes the formation of the pseudo-epiglottis. In a study from Maclean et al. in 2011 with 24 included patients, the pseudo-diverticulum and a pseudo-epiglottis could not be associated with self-reported dysphagia [14]. However, in this study an association between closure technique and a pseudo-diverticulum or dysphagia was not found.

Our retrospective study has its limitations. Dysphagia could not be standardised because not all included patients filled in the SWAL-QOL questionnaire after laryngectomy. In the group of patients without a completed SWAL-QOL questionnaire, information about their swallowing function was collected from the medical files, which results in less precise measurements. Because of this, it was impossible to quantify the severity of dysphagia and therefore differences in dysphagia can be assumed. Furthermore, we did not correct for other causes of dysphagia, for instance tumour recurrence, pharyngeal dysmotility or stricture formation. This could be an explanation for the results of no statistically proved difference between the formation of a pseudo-diverticulum and postoperative dysphagia. In a future study, it would be beneficial to use information about dysphagia from validated questionnaires, so more stringent criteria could be used to define postoperative dysphagia. Furthermore, there could be disparity in the size of the measured pseudo-diverticulas. It is not possible to take barium swallow radiographs of different patients from exactly the same angles. These slightly different angles could have had influenced the measurements of the depth of the pseudo-diverticula. Strength of this study is a well-defined cohort of consecutive patients undergoing laryngectomy surgery in the VU Medical Centre. Presence and size of the pseudo-diverticulum was assessed blinded for the type of closure and the patient records were collected independently of the outcome.

## Conclusion

In our retrospective study, vertical closure of the neopharynx in laryngectomy surgery showed a higher risk for developing a pseudo-diverticulum than “T”-shaped closure. Furthermore, depth of the pseudo-diverticulum was deeper in the vertical closure group compared to the “T”-shaped closure group. In the group of patients with a pseudo-diverticulum, dysphagia was more often reported than in the group of patients without a pseudo-diverticulum. Type of closure is not associated with more or particular postoperative complications. Concluding, we recommend applying “T”-shaped closure instead of vertical closure in laryngectomy surgery.
